# A primary pulmonary artery sarcoma masquerading pulmonary embolism: a case report and literature review

**DOI:** 10.1186/s12959-023-00578-0

**Published:** 2024-01-04

**Authors:** Zhiyue Liu, Lili Fan, Shichu Liang, Zhong Wu, He Huang

**Affiliations:** 1 Department of Cardiology, West China Hospital, Sichuan University, No. 37 GuoXue Alley, Chengdu, 610041 China; 2 Department of Respiratory and Critical Care Medicine, West China Hospital, Sichuan University, Chengdu, China; 3 Department of Cardiovascular Surgery, West China Hospital, Sichuan University, No. 37 GuoXue Alley, Chengdu, 610041 China

**Keywords:** Contrast echocardiography, Pulmonary artery sarcoma, Pulmonary embolism

## Abstract

**Background:**

Primary pulmonary artery sarcoma (PAS) is an extremely rare malignant tumor with a poor prognosis. The clinical manifestations of PAS are diverse, including dyspnea, chest pain, cough, and hemoptysis. The poor prognosis is often due to delayed diagnosis caused by similarity in imaging findings with pulmonary thromboembolism (PTE). These cues of diagnosis include the “wall eclipsing sign”, lobulated bulging margins, gadolinium enhancement during MRI imaging, and FDG uptake during PET/CT imaging. However, there are still many misdiagnoses.

**Case presentation:**

This article reports a woman of reproductive age presenting with a pulmonary artery mass. The computed tomographic pulmonary angiography and positron emission tomography/computed tomography did not show obvious signs of pulmonary artery sarcoma, however, contrast-enhanced echocardiography showed moderate perfusion, which helped differentiate between pulmonary artery sarcoma and pulmonary artery thrombosis, leading to timely surgical treatment.

**Conclusions:**

PAS is a rare form of cancer that can occasionally be visually similar to PTE on radiographic images. Early diagnosis of PAS is of vital importance to the prognosis of the patients. There are several visual cues that can help differentiate between the two conditions. Additionally, contrast-enhanced echocardiography provides additional information on tumor perfusion, offering another effective approach for a prompt and accurate diagnosis.

## Background

Pulmonary artery sarcoma (PAS) is a rare and malignant tumor that develops within the inner or middle layer of the pulmonary artery, with an estimated incidence rate ranging from 0.001–0.03% [1]. The survival period of PAS approximately 1.5 months for those who did not undergo surgery timely. Currently, surgical resection remains the main treatment for PAS. Early and aggressive surgical removal plays a crucial role in extending the patient’s lifespan [2, 3]. However, the lack of specific symptoms associated with PAS often results in misdiagnosis as pulmonary thromboembolism (PTE), leading to delays of appropriate treatment for PAS [4]. Therefore, early, and accurate diagnosis of PAS is of utmost importance in determining the prognosis for patients.

Previous study indicated that positron emission tomography/computed tomography (PET/CT) is the preferable method for distinguishing between PAS and PTE. In cases where the tumor lesions show strong 18 F-FDG uptake while PTE appears as negative on PET/CT imaging [5]. We present a case of a reproductive-aged woman with a mass in the pulmonary artery. Both computed tomographic pulmonary angiography (CTPA) and PET/CT did not reveal any clear signs of primary PAS. However, contrast echocardiography demonstrated moderate perfusion, aiding in the differentiation between PAS and PTE. This information guided the decision for timely surgical intervention.

## Case presentation

A 34-year-old female with a history of oral contraceptives use was admitted to local hospital due to exertional dyspnea and experiencing syncope for the past 8 days. The patient reported dyspnea while climbing stairs, along with intermittent episodes of transient loss of consciousness lasting approximately 10 s. There were no reported concomitant symptoms of palpitations, chest tightness, headache, vertigo, vomiting, or persistent dyspnea. The echocardiography at the local hospital indicated PTE.

Therefore, she was transferred to our hospital for further therapy. Given her medical history and the possibility of PTE, we initiated anticoagulation therapy with heparin. Further investigations revealed the following results: D-dimer was 1.60 mg/l FEU (reference range < 0.05 mg/l FFU). Tumor markers showed elevated levels of aldehyde dehydrogenase at 32.7 ng/ml (reference range < 20.4 ng/ml). Anti-nuclear antibodies, anti-neutrophil cytoplasmic antibodies, anti-cyclic citrullinated peptide antibodies, anticardiolipin antibodies, anti-β2 glycoprotein I antibodies, rheumatoid factor, and lupus anticoagulant were all negative.

**Fig. 1:**
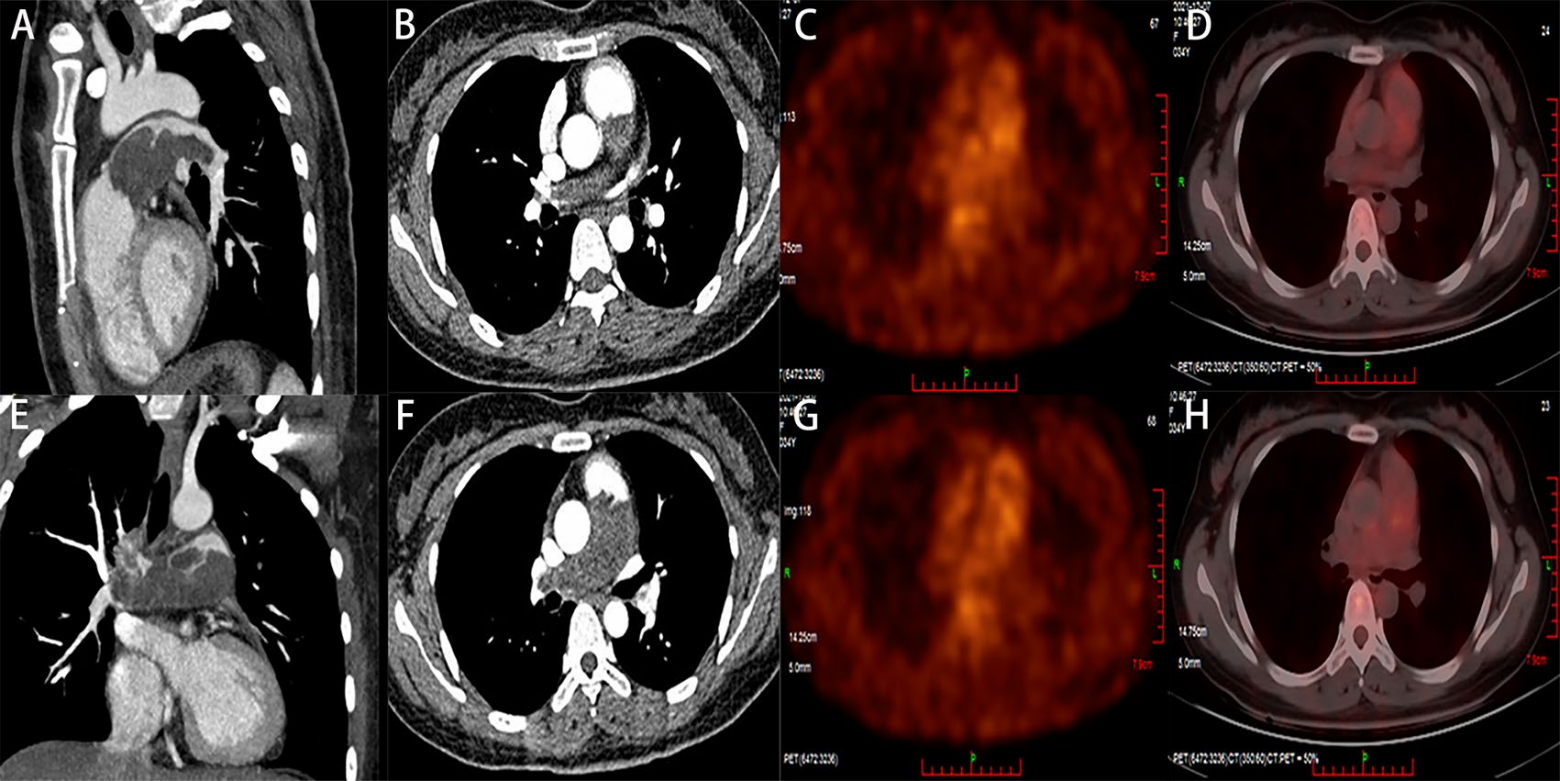
**A**, **B**, **E**, **F** Preoperative CTPA: The dilation of the pulmonary trunk, filling defects in the main pulmonary artery, left and right pulmonary arteries. **C**, **D**, **G**, **H** PET/CT: a slight increase in glucose metabolism of the mass

The CTPA showed filling defects in the main pulmonary artery and its branches (Fig. 1). The ultrasound examination of the limbs yielded normal findings, ruling out deep vein thrombosis.

**Fig. 2:**
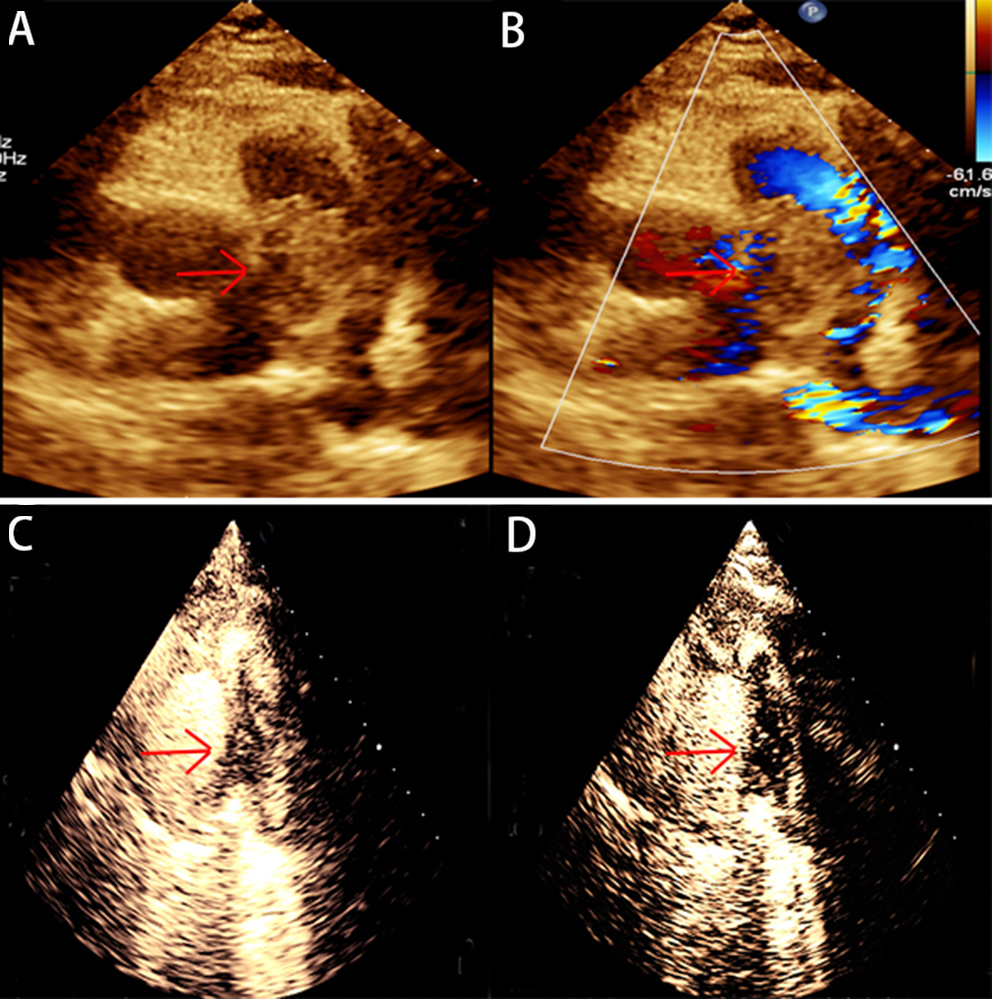
**A**, **B** Preoperative echocardiography: a large irregular hypoechoic mass detected within the main pulmonary artery. No blood flow revealed in the mass. **C**, **D** Contrast-enhanced echocardiography: the contrast agent was unevenly filling within the mass

As the patient currently does not exhibit acute embolic symptoms, there is a need to remain vigilant for in-situ thrombosis caused by malignancy. Therefore, a PET/CT scan has been arranged to evaluate the nature of the occupying lesions, and another echocardiography was conducted to evaluate the efficacy of anticoagulation. The PET/CT scan did not reveal any conclusive signs of malignancy throughout the body. Nevertheless, there was a hypodense lesion in the main pulmonary artery and its branches, along with mild FDG activity (Fig. 1). Echocardiography revealed hypoechoic mass within the main pulmonary artery trunk and its branches (Fig. 2). It also indicated the presence of severe pulmonary hypertension, as well as enlarged right heart chambers with normal systolic function. However, the contrast-enhanced echocardiography displayed a moderate perfusion of the mass (Fig. 2).

**Fig. 3:**
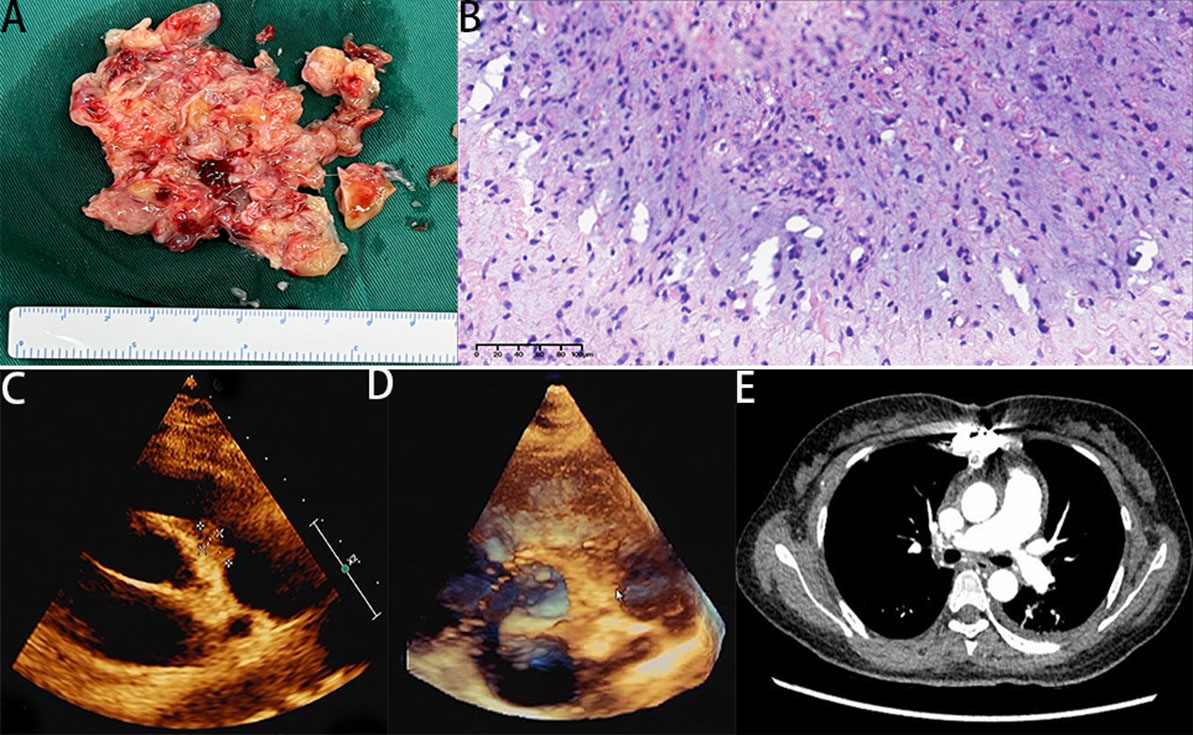
**A** A mass was observed within the main pulmonary artery and the branches of the left and right pulmonary arteries. The mass appeared soft and dark in color. **B** Postoperative pathology revealed a spindle cell tumor, which was diagnosed as a sarcoma based on the combination of immunohistochemistry and genetic testing. **C**, **D** Postoperative echocardiography: there was an irregular hypoechoic mass measuring approximately 16 × 9 mm detected at the level of the pulmonary valve annulus, which was closely related to the pulmonary artery valve. **E** Postoperative CTPA: membranous structures were observed in the main pulmonary artery and left pulmonary artery

During hospitalization, she experienced recurrent episodes of syncope, resulting in unconsciousness, unresponsiveness, with a heart rate of 45 bpm, respiratory rate of 10 breaths per minute, and undetectable oxygen saturation level. Due to the suspected presence of a tumor, emergency surgery was performed. During the procedure, the occupying lesions was found within the main, left and right pulmonary artery. These masses exhibited a soft texture and dark color, with unclear boundaries between the masses and the pulmonary vessel wall (Fig. 3). Furthermore, the masses extensively infiltrated the pulmonary valve and the pulmonary vessel wall. After the resection, due to severe myocardial edema in the patient, sternum closure would severely compress the right heart. The chest incision is closed with multiple layers of antibacterial dressings, and the patient is sent to the intensive care unit for secondary monitoring. Sternum closure will be performed at an elective time.

The postoperative pathological examination confirmed the presence of a spindle cell tumor (Fig. 3). Further immunohistochemical and genetic testing demonstrated the diagnosis of a malignant sarcoma. The specific subtype was identified as intimal sarcoma with FNCLCC grade 3 classification. Immunohistochemistry results exhibited positive expression of MDM2, CDK4, P16, with focal desman positivity, focal EMA positivity, focal TLE-1 positivity, and a Ki-67 proliferation index of 30%. FISH testing revealed amplification of the MDM2 and FRS2 genes, but no amplification of the CDK4 gene.

The patient recovered well after surgery and discharged. The postoperative echocardiography revealed the presence of an irregular hypoechoic mass measuring approximately 16 × 9 mm at the level of the pulmonary valve annulus. This mass was closely associated with the pulmonary artery valve. Additionally, the postoperative CTPA showed the presence of membranous structures within the main pulmonary artery and left pulmonary artery.

Unfortunately, this patient experienced syncope again and passed away 2 months after the surgery, likely attributed to two primary factors. Firstly, immediate closure of the chest wall was not feasible following the surgery due to tissue swelling. Once the inflammation subsided, the chest wall was subsequently closed again. Secondly, as the tumor had spread to the pulmonary valve, complete separation was not achievable, resulting in a relapse shortly.

## Discussion

PAS typically presents with an indolent onset, and its clinical symptoms resemble those of PTE. Common manifestations of PAS include exertional dyspnea, chest pain, cough, hemoptysis, and fatigue [6]. The treatment for PTE is anticoagulation or thrombolytic therapy. However, for the treatment of PAS, there is no standard treatment plan. Surgical resection is the main treatment method, and the possibility of surgery depends on factors such as the patient’s cardiopulmonary reserve, tumor location, and presence of distant metastasis. It has been reported that approximately 47% of patients with primary PAS were initially misdiagnosed as having PTE before undergoing surgery. Furthermore, this misdiagnosis led to a delay in surgical intervention for about 39% of these patients [7]. Therefore, achieving a timely and accurate diagnosis of PAS is crucial for ensuring the best possible prognosis for patients with PAS.

Compared to PTE, patients with PAS might exhibit additional symptoms such as fever, anemia, weight loss, increased erythrocyte sedimentation rate, and absence of hypercoagulability [8]. Previous studies have indicated that levels of D-dimer in patients with PAS were usually within the normal range. Therefore, the presence or absence of elevated D-dimer levels may serve as an important indicator for differentiating between PAS and PTE [9]. Other factors that can help distinguish between PTE and PAS include the patient’s history of contraceptive use, and hypercoagulability due to various reasons. Additionally, the presence of deep vein thrombosis is also considered a contributing factor to the development of PTE. The elevated D-dimer level may be associated with the formation of the local thrombus [10]. Nearly 5% patients had concurrent large thrombus burden surrounding the tumor [7]. Furthermore, the release of procoagulant substances by tumor cells, such as mucins and coagulation factors, can induce a hypercoagulable state and secondary fibrinolysis hyperactivity, which might also contribute to the elevated D-dimer levels. Therefore, an elevated D-dimer level alone cannot directly exclude the diagnosis of primary PAS. In this case, the patient had only mild elevated D-dimer levels which could be a potential distinguishing element between PAS and PTE.

**Fig. 4:**
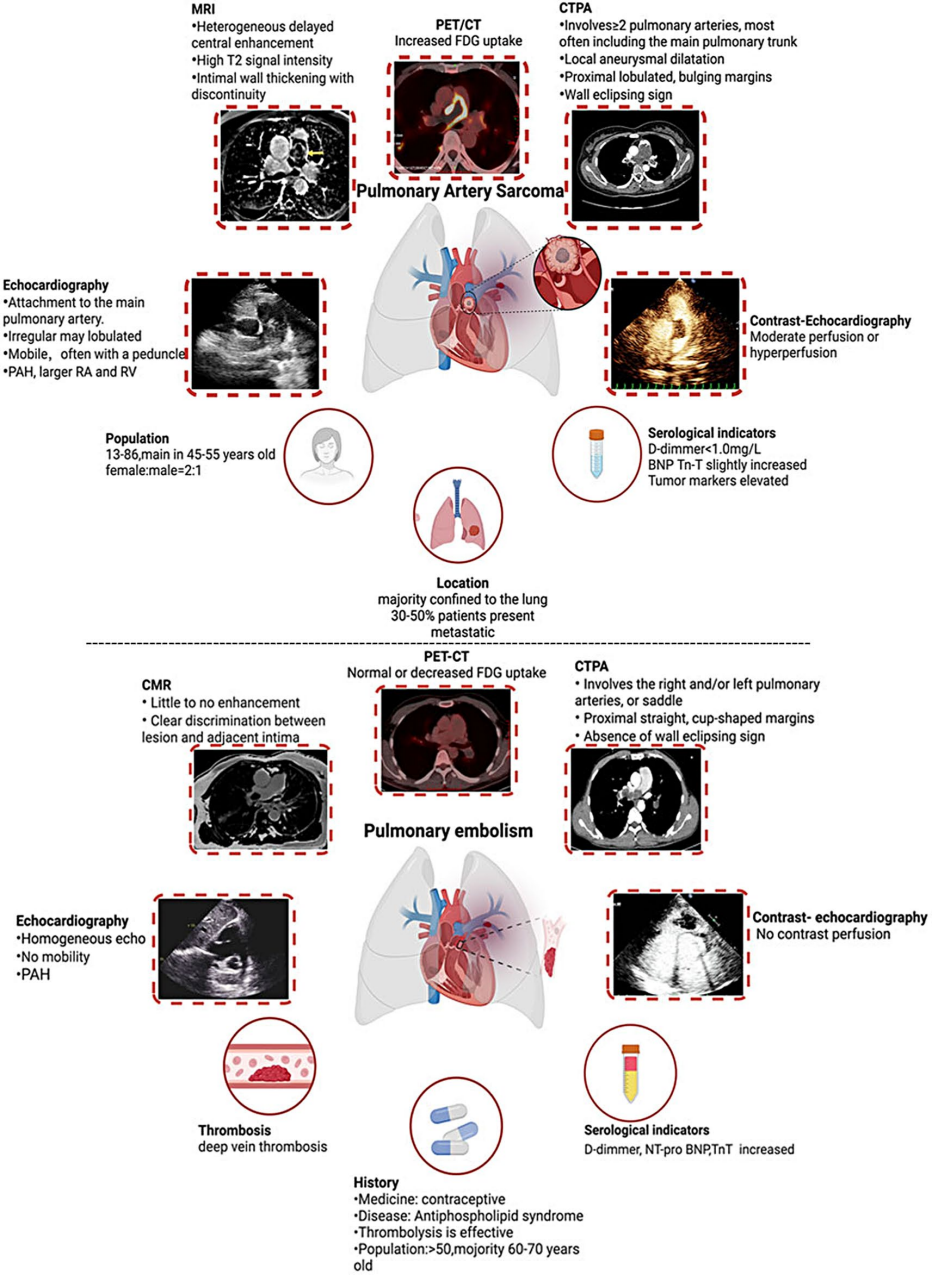
The multimode image characteristics of PAS and PTE (note: The CMR of PAS reprinted from European Heart Journal. Nijjar PS, Iqbal FM, Alraies MC, Valeti US, Tadavarthy SM. Primary pulmonary artery sarcoma masquerading as pulmonary embolism: role of cardiac MRI. Eur Heart J. 2016;37(18):1479)

Imaging examinations also play a crucial role in the diagnosis of PAS (Fig. 4). PAS is primarily manifested on CTPA as a larger mass located in the main pulmonary artery, left or right pulmonary artery, or even the right ventricular outflow tract [11]. The tumor exhibits irregular margins and may display lobulations or septations. It often causes dilation of the proximal pulmonary artery and adjacent branch vessels. Additionally, aneurysmal dilation in the distal arteries may be observed, resembling grape-like nodules within the lung [11]. Therefore, the location of the lesions can be important for differentiating PAS from other conditions. Previous studies have reported that 85% of PAS cases involve the main pulmonary artery, 71% involve the right pulmonary artery, 65% involve the left pulmonary artery, 32% involve the pulmonary valve, and 10% involve the right ventricular outflow tract [12]. Also, some PAS may invade the lungs or mediastinum [13]. In contrast, PTE often occurs in the right lung, bilateral lower lungs, and peripheral pulmonary arteries, which rarely affects the main pulmonary artery or other areas.

The differences in morphological characteristics also hold significance in distinguishing between PAS and PTE, which were summarized in Fig. 4 and Table 1. Due to the localized accumulation, expansion, and infiltration of tumor tissue into the walls of the pulmonary artery, PAS appears as a massive embolus on CTPA, almost completely occupying the entire lumen of the pulmonary arteries. The tumor mass aligns with the course of the pulmonary vascular tree, and the free end convex to the blood flow, giving rise to the term “wall eclipsing sign”, which is a characteristic sign of PAS [14]. However, in this case, no evident “wall eclipsing sign” was observed on the CTPA, which can lead to a misdiagnosis of PTE. In contrast, in cases of PTE patients, the emboli are unlikely to completely block the main pulmonary artery due to the hemodynamic forces and activation of the body’s fibrinolytic system. As a result, they often manifest as proximal filling defects that appear flat, or cup shaped. In addition, lobulated PAS often creates sharp angles with the vessel wall, whereas chronic PTE tends to form blunt angle [15]. These morphological differences can aid in differentiating between PAS and PTE.

Magnetic resonance imaging (MRI) offers high-resolution capabilities and can aid in distinguishing between PAS and PTE by assessing flow and gradients throughout the pulmonary artery vasculature [16]. Regarding the enhancement patterns observed in MRI, PAS commonly presents with aneurysm- or grape-like distal structures exhibiting heterogeneous enhancement [17]. This unique imaging marker is indicative of PAS. The level of enhancement is also associated with tumor differentiation [16]. PAS tumors typically exhibit a varied pattern of delayed enhancement, with time-signal intensity curves that gradually increase over time. Additionally, they tend to have higher T2 signal intensity compared to PTE [5]. However, PAS patients often experience respiratory difficulties, and the prolonged breath-holding time limits the practicality of using MRI in such cases. As a result, the clinical utilization of MRI in cases involving tumors was infrequent in the past.

In PET/CT examinations, PTE often presents as radiotracer sparsity or defects, which is important in distinguishing between PAS and PTE [18, 19]. What is more, a single PET/CT examination can provide information on the metabolic activity of the pulmonary artery mass while also offering an overview of the patient’s systemic condition. It can also help determine the presence or absence of distant metastasis, allowing for a comprehensive evaluation of malignant tumors in a single session [20]. Studies have elucidated that the maximum standardized uptake value (SUVmax) can be used to differentiate between PAS and PTE. Ito K et al. [21]demonstrated that the mean SUVmax of PAS (7.63 +/- 2.21) was significantly higher than that of PTE (2.31 +/- 0.41). Xi et al. [22]further indicated that, employing a cutoff value of 3.3, the sensitivity, specificity, and accuracy were found to be 98.4%, 96.8%, and 97.8%, respectively. These studies highlight the significance of PET/CT in the diagnosis of PAS, nevertheless, these are all single-center studies with limited samples. It should be noted that in some cases, the ^18^F-FDG uptake may be low, resulting in negative findings on PET/CT and potentially leading to a misdiagnosis of PTE. [23] The low ^18^F-FDG uptake can be attributed to the limited presence of tumor cells and an abundance of mucinous tissue [23]. Additionally, tumor necrosis, hemorrhage, calcification [24], or chronic thrombosis [25]can also contribute to the low uptake of ^18^F-FDG in tumors. Therefore, PET/CT may not be the most optimal screening tool, particularly due to its cost and accessibility [26]. In cases where there is a strong suspicion of PAS, a negative PET/CT result cannot entirely exclude the diagnosis. In this case, the ^18^F-FDG uptake is low, which confounded the diagnosis of PAS [27]. As a result, a subsequent contrast echocardiography was performed to assess the perfusion of the mass and provide further valuable information.

Transthoracic echocardiography is one of the essential methods for diagnosing PAS, which allows for dynamic observation of the tumor’s location, size, shape, activity, spatial structure, and adjacent relationships, providing a preliminary assessment of the tumor’s benign or malignant nature and its degree of infiltration [12]. Color Doppler can display the bloodstream signals within the tumor.

Contrast-enhanced echocardiography demonstrated excellent sensitivity and specificity in distinguishing between thrombi and PAS [28, 29]. Contrast-enhanced imaging allows for dynamic observation of the complete morphology of PAS, including the width of the attached base, presence of a stalk, and mobility, with the assistance of contrast agents. By assessing the contrast perfusion within the lesion, it helps to differentiate between tumors and thrombi, as well as evaluate the extent of infiltration into the branch arteries. Additionally, it enables simultaneous assessment of pulmonary artery hypertension caused by tumor obstruction. On the other hand, contrast-enhanced ultrasound imaging during PTE shows no contrast perfusion within the lesion. Furthermore, the contrast agents used in echocardiography are radiation-free and less likely to cause allergic reactions, making them a safer option for patients. They can be excreted through the alveoli, resulting in minimal impact on renal function. Additionally, they have high repeatability and can be administered multiple times if necessary [30].

**Table 1:** Comparing PAS and PTE in clinical imaging

	PAS	PTE
CTPA	• Occupying the entire lumen of the pulmonary arteries • “Wall eclipsing sign” • Sharp angles with the vessel wall	• Appear flat, or cup shaped • Blunt angle with the vessel wall
MRI	• Aneurysm- or grape-like distal structures exhibiting heterogeneous enhancement • Delayed enhancement • Higher T2 signal intensity • Artery wall thickening	• No enhancement and delayed enhancement • Clear demarcation with artery wall
PET/CT	High ^18^F-FDG uptake	Low/no ^18^F-FDG uptake
TTE	• Expansive growth with raised border • Medium-low echo, with an uneven internal echo • Slightly mobile • Color doppler display obvious blood flow signal inside the cyst	• Regular morphology • Uniformly hypoechoic • Immobile • Distal end to proximal end • No obvious blood flow signal inside the cyst
Contrast-TTE	Moderate-high perfusion	Low perfusion

CTPA computed tomographic pulmonary angiography; MRI magnetic resonance imaging; PET/CT positron emission tomography/computed tomography; TEE trans-thoracic echocardiography

## Conclusions

PAS is a rare form of cancer that can occasionally be visually similar to PTE on radiographic images. Early diagnosis of PAS is of vital importance to the prognosis of the patients. There are several visual cues that can help differentiate between the two conditions. These cues include the “wall eclipsing sign”, lobulated bulging margins, gadolinium enhancement during MRI imaging, and FDG uptake during PET/CT imaging. Additionally, contrast-enhanced echocardiography provides additional information on tumor perfusion, offering another effective approach for a prompt and accurate diagnosis.

## Data Availability

The data presented in this study are available on reasonable request from the corresponding author.

## References

[1] Lashari BH, Kumaran M, Aneja A, Bull T, Rali P (2022). Beyond clots in the pulmonary circulation: pulmonary artery tumors mimicking Pulmonary Embolism. Chest.

[2] Song W, Zhong Z, Liu S (2022). Complete resection of a pulmonary artery sarcoma involving the pulmonary valve and right ventricle outflow tract: a case report. Eur Heart J Case Rep.

[3] Hu HM, Li YD, Wei CW, Liu Y, Lv XZ, Yang YH (2023). Pulmonary artery sarcoma: an unexpected settler in the right ventricular outflow tract. J Cardiothorac Surg.

[4] Ropp AM, Burke AP, Kligerman SJ, Leb JS, Frazier AA (2021). Intimal Sarcoma of the great vessels. Radiographics.

[5] Kronzer E, Robinson SI, Collins DA, McBane RD (2021). Primary pulmonary artery sarcoma versus pulmonary thromboembolism: a multimodal imaging comparison. J Thromb Thrombolysis.

[6] Parish JM, Rosenow EC, Swensen SJ, Crotty TB (1996). Pulmonary artery sarcoma. Clinical features. Chest.

[7] Bandyopadhyay D, Panchabhai TS, Bajaj NS, Patil PD, Bunte MC (2016). Primary pulmonary artery sarcoma: a close associate of Pulmonary Embolism-20-year observational analysis. J Thorac Dis.

[8] Zhang S, Zhang Y, Liu M, Tao X, Xie W, Wan J, Zhai Z (2021). Radiological, histopathological findings, and clinical outcome of pulmonary artery sarcoma. Pulm Circ.

[9] Guo W, Zhang W, Huang X, Liang Y, Gan H, Chen D, Liu S, Ma H (2014). [Clinical characteristics of 9 patients with pulmonary artery sarcoma]. Zhonghua Xin xue guan bing za zhi.

[10] Tuft C, Maheepala K, Raguparan A, Naeem A, Lodh S, Lindstrom S (2022). Pulmonary artery sarcoma: an important mimic of pulmonary embolism-case reports and literature review. Respirol Case Rep.

[11] Liu M, Luo C, Wang Y, Guo X, Ma Z, Yang Y, Zhang T (2017). Multiparametric MRI in differentiating pulmonary artery sarcoma and pulmonary thromboembolism: a preliminary experience. Diagn Interv Radiol.

[12] Al-Mehisen R, Al-Halees Z, Alnemri K, Al-Hemayed W, Al-Mohaissen M (2019). Primary pulmonary artery sarcoma: a rare and overlooked differential diagnosis of Pulmonary Embolism. Clues to diagnosis. Int J Surg Case Rep.

[13] Leitman EM, McDermott S (2019). Pulmonary arteries: imaging of Pulmonary Embolism and beyond. Cardiovasc Diagn Ther.

[14] Gan HL, Zhang JQ, Huang XY, Yu W (2013). The wall eclipsing sign on pulmonary artery computed tomography angiography is pathognomonic for pulmonary artery sarcoma. PLoS ONE.

[15] Wittram C, Maher MM, Yoo AJ, Kalra MK, Shepard JA, McLoud TC (2004). CT angiography of Pulmonary Embolism: diagnostic criteria and causes of misdiagnosis. Radiographics.

[16] von Wyler MC, Chan EY, Reardon MJ (2019). Imaging and Surgical Treatment of Primary Pulmonary Artery Sarcoma. Int J Cardiovasc Imaging.

[17] Liu MX, Ma ZH, Jiang T, Guo XJ, Yu FF, Yang YH, Zhai ZG (2018). Differential diagnosis of Pulmonary Artery Sarcoma and Central Chronic Pulmonary Thromboembolism using CT and MR Images. Heart Lung Circ.

[18] Pan B, Wang SC, Chen ZK, Chen X (2021). Primary pulmonary artery sarcoma with intrapulmonary metastases based on PET/CT imaging: a case report and literature review. Ann Palliat Med.

[19] Ren J, Li H, Zhang Q, Liu E, Zeng B, Huang Y, Wang L, Jiang L (2022). Clinical utility of (18)F-FDG PET/CT imaging in patients with pulmonary artery sarcoma. EJNMMI Res.

[20] Hmelik S, Dobrenić M, Huić D (2018). F-18 FDG PET/CT in pulmonary artery sarcoma: clinical vignette. Nucl Med Rev Cent East Eur.

[21] Ito K, Kubota K, Morooka M, Shida Y, Hasuo K, Endo H, Matsuda H (2009). Diagnostic usefulness of 18F-FDG PET/CT in the differentiation of pulmonary artery sarcoma and Pulmonary Embolism. Ann Nucl Med.

[22] Xi XY, Gao W, Gong JN, Guo XJ, Wu JY, Yang YH, Yang MF (2019). Value of (18)F-FDG PET/CT in differentiating malignancy of pulmonary artery from pulmonary thromboembolism: a cohort study and literature review. Int J Cardiovasc Imaging.

[23] Koike T, Iwata H, Hirose K, Minamino T (2023). A case report of pulmonary artery intimal sarcoma negative for 18F-FDG mimicking pulmonary thromboembolism. Eur Heart J Case Rep.

[24] Lee DH, Jung TE, Lee JH, Shin DG, Park WJ, Choi JH (2013). Pulmonary artery intimal sarcoma: poor 18F-fluorodeoxyglucose uptake in positron emission computed tomography. J Cardiothorac Surg.

[25] Jiang M, Zheng J, Chen P, Zhou W (2021). Primary pulmonary artery sarcoma concomitant with chronic thromboembolism: role of 18F-FDG PET/CT. Eur Heart J Cardiovasc Imaging.

[26] Le Roux PY, Robin P, Delluc A, Tardy B, Abgral R, Couturaud F, Reffad A, Le Gal G, Salaun PY (2015). Performance of 18F fluoro-2-désoxy-D-glucose positron emission tomography/computed tomography for the diagnosis of venous thromboembolism. Thromb Res.

[27] Murphy CG, Goldstein JM, Besharati S, Kobsa S, Salvatore MM, Rosenzweig EB, Ingham M, Del Portillo A, Takeda K, Chandra S (2022). A 52-Year-old man with chest Pain and Dyspnea. Chest.

[28] Sun T, Lu GL, Ma LC, Huang JZ, Xie SB (2022). Multimodal echocardiography in the diagnosis of masses localized to the proximal portions of pulmonary arteries. Int J Cardiovasc Imaging.

[29] Jiang W, Liu M, Guo X, Li J, Gong J, Yang M, Liu Y, Gu S, Li Y, Yang Y, et al. Echocardiographic characteristics of Pulmonary Artery Intimal Sarcoma: comparison with CTPA. Heart Lung Circ; 2023. 10.1016/j.hlc.2023.05.013 37355431

[30] Porter TR, Mulvagh SL, Abdelmoneim SS, Becher H, Belcik JT, Bierig M, Choy J, Gaibazzi N, Gillam LD, Janardhanan R (2018). Clinical applications of Ultrasonic Enhancing agents in Echocardiography: 2018 American Society of Echocardiography Guidelines Update. J Am Soc Echocardiogr.

